# Occurrence of Potential Bacterial Pathogens and Their Antimicrobial Susceptibility Patterns Isolated from Herbal Medicinal Products Sold in Different Markets of Gondar Town, Northwest Ethiopia

**DOI:** 10.1155/2016/1959418

**Published:** 2016-05-19

**Authors:** Abdela Yesuf, Yitayih Wondimeneh, Teklay Gebrecherkos, Feleke Moges

**Affiliations:** ^1^Department of Medical Laboratory, Debark Hospital, P.O. Box 196, Debark, Ethiopia; ^2^School of Biomedical and Laboratory Sciences, College of Medicine and Health Sciences, University of Gondar, P.O. Box 196, Gondar, Ethiopia

## Abstract

*Background*. The World Health Organization estimates that about 80% of the world's population uses herbal medicine to treat various illnesses as means of primary healthcare. However, during preparation, herbal plants may be exposed to contamination by potential pathogens, and this may lead to infections. The aim of this study was to determine bacterial contamination of herbal medicinal products and to assess the antibiotic susceptibility pattern of the isolated bacteria.* Methods*. A cross-sectional study was conducted from January 1 to May 25, 2013, at Gondar Town. A total of 55 samples used as oral, local, and intranasal routes of administration were collected from the herbalists.* Results*. In the present study the total aerobic bacterial count ranges from zero to 2.41 × 10^9^ CFU/g with mean count of 1.99 × 10^8^ CFU/g or mL while the total coliform count showed an average of 1.05 × 10^8^ CFU/g or mL with a range of zero to 2.1 × 10^9^ CFU/g. The most common bacteria isolated were* Bacillus* spp. followed by* Enterobacter* spp.,* Shigella dysenteriae*, and* Salmonella* spp. Multiple drug resistance was not uncommon and it was found that 125 (83.4%) of the isolates were resistant to two or more antibiotics.* Conclusion*. Herbal medicinal preparations were highly contaminated with pathogenic microorganisms with high microbial load. Most of the isolates have multiple drug resistance. Using those contaminated herbal medicines may lead to infection of other health related risks. Therefore, this warrants urgent training of herbalists and management scale-up for quality and safety of medicinal plants.

## 1. Introduction

The use of herbal medicine is generally increasing worldwide [1]. In many developed countries, 70–80% of the population still depends upon herbal drugs for their health care [1–3]. Similarly in developing countries such as Africa, up to 80% of the population relies on herbal medicine as a major source of therapy [1, 3, 4].

Although the World Health Organization (WHO) has advocated the integration of herbal medicinal products (HMPs) into the primary health care system of developing countries, safety issues related to herbal drug preparations continue to be ignored by the herbalist [5]. So the safety of herbal products became a major concern in public health [6]. This is because microorganisms of various kinds are normally adherent to leaves, stem, flowers, seeds, and roots from which herbal medicine can be prepared and potential pathogens may also be introduced during harvesting, handling [6, 7], open-air drying, preserving, manufacturing [6], and use of contaminated materials for storage [7, 8]. According to some reports, the consumers may possibly fall into illness because of taking herbs incriminated with pathogenic microorganisms [8] and sometimes the presence of antibiotic resistant microbial isolates in the HMPs will lead to transfer of antibiotic resistance strains to consumers [9]. Different studies that have been performed on herbal medicinal products revealed the presence of bacterial pathogens with multiple drug resistance [10].

In Ethiopia, due to the cultural acceptability of healers, the relatively low cost of traditional medicine, and limited access to modern health facilities [11] up to 80% of the population uses traditional medicine [11, 12]. Ethiopia has high diversity of plant species most of which are used in the preparation of herbal medicinal products and it is one of the six African countries where about 60% of the plants are said to be indigenous with their healing potential [13]. The growing and unhygienic use of herbal medicinal products has become a major concern in public health [6]. However, in Ethiopia particularly in the study area, there is no sufficient data which addresses the bacteriological quality of herbal medicinal products. Hence the aim of this study was to assess the occurrence of potential bacterial pathogens and their antimicrobial susceptibility patterns isolated from herbal medicinal products sold in different markets of Gondar Town, Northwest Ethiopia.

## 2. Materials and Methods

### 2.1. Study Area, Design, and Period

A cross-sectional study was conducted from January 1 to May 25, 2013, at Gondar Town. Gondar is located in the Northwestern part of Ethiopia, about 740 km far from Addis Ababa, the capital city of Ethiopia. The town has an estimated area of 41.27 square Km, with altitude of 2200 meters above sea level, having a total population of more than 252,537 [14].

### 2.2. Source of Population

All types of herbal medicinal products with oral, local, and intranasal route of administration have been sold at different markets of Gondar Town.

### 2.3. Study Subjects

All types of herbal medicinal products prepared by the 20 herbalists of Gondar Town who have been enrolled in association of traditional medicine named “Zeria-Biruk Association of Traditional Medical Practitioner” were the study subjects.

### 2.4. Sample Size and Sampling Technique

In the present study, a total of 55 (13 liquid and 42 solid) samples were taken from all herbalists. From each herbalist herbal medicinal products prepared for oral, local (body lotion), and inhalation use were considered. All orally, locally, and intranasal consumed powder form preparations and all liquid samples were purchased and included in the study. Route of administration of the herbal traditional medicine can be mainly in oral, body lotion, and inhalation type and the present study gives more attention to those herbal medicinal products.

*Inclusion Criteria*. Those powder and liquid preparations of herbal medicinal products administered orally, locally, and intranasally without further processing were included in the study. 

*Exclusion Criteria*. Those powder form herbal medicinal products with further processing and other routes of administration were excluded in the study.

### 2.5. Data Collection, Handling, and Transporting of Specimens

A total of 55 different herbal (42 powder and 13 liquid) preparations were purchased randomly from identified herbalists in Gondar Town. Using aseptic techniques, from each liquid herbal preparation about 5 mL of sample was collected in a sterile screw-capped test tube and about 3 to 10 g of solid (powder) sample was also collected by using labeled wide-necked test tubes. All the samples were transported to the Medical Microbiology Laboratory of Gondar University Hospital with cold box within one hour of collection. Liquid specimens were refrigerated at 4°C till processing.

### 2.6. Isolation and Identification of Bacteria

Liquid samples were homogenized by mixing vigorously and transferred 1 mL sample to 9 mL sterile saline. At the same time, the liquid solution was prepared by mixing 1-gram powder with 9 mL of tryptone soy broth as a stock solution and then serial dilution was made to get appropriate dilution. All microbiological analyses were carried out in triplicate according to the standard [15–17]. By using a calibrated micropipette, 100 *μ*L volume of 10^−3^, 10^−5^, and 10^−7^ was measured and dispensed by glass rod spreader to standard plate count and violet red bile agar media. Liquid preparations were also cultured on blood agar, MacConkey agar, and Salmonella Shigella agars and then incubated at 35–37°C for 18 to 24 hours.

At the end of the incubation period, the colonies on standard plate count media and violet red bile agar media were enumerated by using surface plate agar method using colony counter for total aerobic count and total coliform bacteria count, respectively. By multiplying the average number of colonies with dilution factor, it was calculated and reported as a colony forming unit per gram (CFU/g) of sample [16–19]. The obtained CFU/g from 1-gram sample was compared with WHO standard [5, 20]. Pure isolate of bacterial pathogen was preliminarily characterized by colony morphology and gram stain [19]. Bacterial identification was made using biochemical tests, namely, indole, citrate, oxidase, H_2_S production, lysine decarboxylase, lactose fermentation, urea hydrolysis, gas production catalase, the coagulase, mannitol fermentation, and novobiocin susceptibility test. A standard biochemical procedure was also used for full identification of gram positive and gram negative bacteria [21].

### 2.7. Antimicrobial Susceptibility Testing

The antibiotic susceptibility test was performed by using disc diffusion method recommended by the Clinical and Laboratory Standards Institute (CLSI) guidelines [22] on Mueller-Hinton agar plate (Oxoid Ltd., Basingstoke, Hampshire, UK). The antibiotic discs and their concentration were amoxicillin (AML, 10 *μ*g), chloramphenicol (C, 10 *μ*g), cloxacillin (CXL, 5 *μ*g), cotrimoxazole (SXT, 50 *μ*g), ampicillin (AMP, 10 *μ*g), ceftriaxone (CRO, 30 *μ*g), gentamicin (CN, 10 *μ*g), penicillin (PEN, 5 *μ*g), nitrofurantoin (F, 300 *μ*g), norfloxacin (N), and erythromycin (E, 5 *μ*g). The inoculum was standardized by transferring pure colonies of the test organism into 5 mL of tryptone soy broth. The broth then incubates for three hours at 35–37°C to allow the growth of test organism and their inoculums sizes were compared with the turbidity of 0.5 McFarland standard [10]. A sterile cotton swab was dipped into the suspension and excess suspension was removed by gentle rotation of the swab against the surface of the tube. Before placing the antimicrobial disc, the swab with the bacterial suspension was distributed evenly over the entire surface of Mueller-Hinton plates [10]. The plates were incubated at 37°C for 18–24 hours. The diameter of the zone of inhibition was measured and interpreted using standard chart as sensitive, intermediate, and resistant [22]. The reference strains used as control were* Escherichia coli* (ATCC 25922) and* Staphylococcus aureus* (ATCC 25923).

### 2.8. Data Analysis

Data was checked for completeness, cleaned manually, entered, and analyzed using SPSS version 20 statistical package. Analysis was made using frequency tables. Pearson's test and odds ratio with 95% CI were used for measures of association and *P* value less than 0.05 was considered as statistically significant.

### 2.9. Ethical Considerations

This research project was conducted after obtaining institutional ethical clearance from School of Biomedical and Laboratory Sciences Ethical Committee. A supporting letter was obtained from Zonal Health Bureau and the chairman of the Association for Herbal Medicinal Plants. Informed consent was also obtained from each study participant. All the information obtained from the study was kept confidential.

## 3. Results

### 3.1. Sociodemographic Characteristics

Among the total of 22 herbalists, 20 (16 males and 4 females) of them were enrolled during the study while the other 2 (both males) refused to participate in the study. The mean age of the herbalists was 41.6 years with minimum and maximum age of 27 and 70 years, respectively. Of the study participants 7/20 (35%) of them were illiterate and 1/20 (5%) of them can only read and write but the rest 12/20 (60%) of the herbalists were literate. 16/20 (80%) of the herbalists were Orthodox and 4/20 (20%) of them were Muslims. Regarding their experience, 13 (65%) of the herbalists have more than 20 years, 2 (10%) have 16–20 years, 4 have (20%) 6–10 years, and 1 (5%) herbalist has 5 years.

### 3.2. Total Aerobic and Total Coliform Count

Among 55 samples analyzed, 5 samples (3 liquid and 2 powder) obtained from 5 different herbalists have no growth and 13 samples (9 powder and 4 liquid) obtained from 6 herbalists have zero coliform count. The total aerobic bacterial count of 55 samples was recorded with minimum count of zero (for samples of no growth) and maximum count of 2.41 × 10^9^ CFU/g with mean count of 1.99 × 10^8^ CFU/g and, except one (6 × 10^4^ CFU/mL, which was liquid), all the aerobic counts were beyond WHO tolerable limit. Total coliform count shows an average of 1.05 × 10^8^ CFU/g or per mL with minimum of zero and maximum of 2.1 × 10^9^ CFU/g (Table 1).

### 3.3. Correlation of Microbial Contaminants with Different Herbal Medicines

A total of 150 strains were isolated from the herbal preparations (Table 2). Of the isolates, 98/150 (65.3%) were gram negatives and 52/150 (34.7%) gram positives. 50 (90.9%) contained pathogenic microorganisms or fecal indicators such as* Escherichia coli*,* Staphylococcus aureus,* and* Pseudomonas aeruginosa*. The organism most commonly isolated from the herbal medicines was* Bacillus* 31 (20.7%) followed by* Enterobacter cloacae* 18 (12%),* Shigella dysenteriae* 13 (8.7%),* Citrobacter* spp. 12 (8%),* Providencia* spp. 12 (8%),* Klebsiella pneumoniae* 11 (7.3%),* Escherichia coli* 10 (6.7%),* Staphylococcus aureus* 10 (6.7%),* Staphylococcus epidermidis* 5 (3.3%), and others (Table 3).

### 3.4. Antimicrobial Susceptibility Patterns of the Isolates

Antimicrobial susceptibility patterns of gram negative and gram positive bacterial isolates from herbal medicinal products in Gondar Town are presented as shown in Tables 4(a) and 4(b). Majority of the isolates were resistant to ampicillin (131) (87.3%), followed by amoxicillin clavulanic acid (95) (63.3%), amoxicillin (92) (61.3%), penicillin (30) (57.7%), and nitrofurantoin (73) (48.7%). However, the lowest rates of resistance were observed in gentamycin (4.7%) followed by vancomycin (8) (5.3%), erythromycin (9) (6.0%), ciprofloxacin (11) (7.3%), and norfloxacin (11) (7.3%) (Table 5).

Multiple drug resistance bacteria were among the most isolated groups. The antimicrobial susceptibility-resistance pattern of the contaminating microorganisms revealed that 9/31* Bacillus* species were resistant to 8 antimicrobial agents (ciprofloxacin, tetracycline, norfloxacin, augmentin, vancomycin, ceftriaxone, gentamicin, and cotrimoxazole) and it was also found that 131 (87.3%) of the isolates were resistant to ampicillin, 95 (63.3%) to amoxicillin clavulanic acid (augmentin), 92 (61.3%) to amoxicillin, and 30 (57.7%) to penicillin. Among bacterial isolates* Pseudomonas aeruginosa* were found to be resistant to at least 5 antibiotics tested while half of the isolates of* Klebsiella ozaenae, Providencia* spp., and* Streptococcus pyogenes* were resistant to 5 or more antibiotics tested (Table 6).

## 4. Discussion

Most of the medicinal plants are prepared in open environment and unhygienic condition which gradually lead to contamination of enteric pathogens having public health importance [20]. In the present study, from 55 herbal medicinal preparations, aerobic bacterial counts were obtained from 50 samples, of which only one has permutable limit of bacterial count according to WHO standard [20]. The other 49 samples were beyond WHO limit with minimum count of 4.6 × 10^5^ CFU/mL and maximum of 2.41 × 10^9^ CFU/g with mean count of 2.15 × 10^8^ CFU/g or mL. The total aerobic bacteria count in the present study is in agreement with the higher counts of aerobic bacteria found in herbal materials which have been studied by Ogunshe and Kolajo in Nigeria [23]. On the contrary, the present study has higher aerobic bacteria count than the study conducted from herbal medicinal preparation by Adeleye et al. at Lagos, Nigeria [24]. The reason why the total bacterial count in our study was higher may be due to the primitive ways of preparation of the plant products, poor environmental sanitation, and storage conditions.

In the present study, a positive coliform count was obtained from 37 samples with a minimum count of 1.9 × 10^4^ CFU/g and maximum count of 2.1 × 10^9^ CFU/g with a mean count of 1.56 × 10^8^ CFU/g or mL. All of the positive coliform counts were values above WHO tolerable limit [20]. In a study conducted by Khattak in Peshawar City, Pakistan, the coliform bacteria were detected and the counts ranged from 1.5 × 10^2^ CFU/g to 1.6 × 10^4^ CFU/g [8]. This high coliform count in the present study may be due to unhygienic practice and most of the herbalists work with their living house which may increase human drug contact.

Among the total 55 samples 23 (41.8%) and from the positive total coliform bacteria count samples 23 (62.2%) show fecal indicator organisms like* Escherichia coli*,* Salmonella*,* Shigella*,* Staphylococcus aureus*, or* Pseudomonas aeruginosa* [7, 9]. Similar studies conducted in Nigeria and Kaduna Metropolis by Esimone et al. [9] and by Danladi et al. [7] showed the presence of fecal indicators organisms in herbs due to unsafe collection, transportation, drying, preparing, storing, and dispensing processes.

The present study showed bacterial isolates like* Bacillus*,* Citrobacter*,* Clostridium*,* Enterobacter*,* Escherichia*,* Klebsiella*,* Pseudomonas*,* Salmonella*,* Shigella*,* Staphylococcus*,* Serratia,* and* Streptococcus*. This report was similar to reports in Nigeria [12, 23, 25–27]. The finding of coliforms like* Escherichia coli*,* Salmonella* spp., and* Shigella dysentery* is very important public health concern that needs urgent need of management of herbal medicinal products to insure their safety and quality issue.

Multiple drug resistance was common in the present study; it was found that 131 (87.3%) of the isolates were resistant to ampicillin, 95 (63.3%) resistant to amoxicillin clavulanic acid (augmentin), 92 (61.3%) to amoxicillin, and 30 (57.7%) to penicillin. Adeleye et al., from Nigeria [24], reported that most of the isolates were resistant to ampicillin, penicillin, cotrimoxazole, and gentamicin. A study conducted in Saudi Arabia showed that bacterial isolates of* Shigella* spp.,* Enterobacter* spp.,* Escherichia coli*,* Staphylococcus* spp., and* Klebsiella* spp. were sensitive to amoxicillin and gentamicin [9]. But the present study showed high level of resistance to amoxicillin. This may be due to the overprescription of amoxicillin in our setup which may act as selective pressure for the growth of amoxicillin resistant strains by suppressing the sensitive strains.

## 5. Conclusion and Recommendation

The present study showed that herbal medicinal preparations sold in the study area were highly contaminated with pathogenic microorganisms with very high microbial load. More than 40% of the samples contain fecal indicator organisms. Multiple drug resistance was not uncommon and it was found that 125 (83.3%) of the isolates were resistant to 2 or more antibiotics tested.

Using those contaminated herbal medicines may have very high health risk due to their biological hazard. This warrants urgent need of management of herbalists or herbal medicinal products to scale up its quality and safety issue. Furthermore, establishing a quality and safety diagnostic service and competency certificate for safe drugs may provide quality and safety initiation between herbalists and good information for the customers.

## Figures and Tables

**Table 1:** Total aerobic and total coliform count of herbal medicinal products sold in different markets of Gondar, Northwest Ethiopia, from April 1 to May 25, 2013.

Sample ID	Total aerobic bacteria count	Total coliform count	Sample ID	Total aerobic bacteria count	Total coliform count	Sample ID	Total aerobic bacteria count	Total coliform count
01A	1.84 × 10^7^	1.5 × 10^7^	07A	1.81 × 10^8^	1 × 10^7^	16C	1.61 × 10^7^	1.12 × 10^7^
01B	0	0	08A	8.1 × 10^6^	7.1 × 10^6^	16D	5.5 × 10^8^	5.1 × 10^8^
01C	1.05 × 10^6^	1 × 10^6^	08B	4.1 × 10^7^	5.1 × 10^6^	16E	5.2 × 10^7^	0
01D	4.9 × 10^7^	3.4 × 10^6^	09A	4.6 × 10^5^	3.2 × 10^5^	16F	1.2 × 10^6^	0
01E	5 × 10^6^	0	09B	4.11 × 10^7^	3.1 × 10^7^	16G	2.4 × 10^8^	3.6 × 10^6^
01F	1.11 × 10^6^	0	10A	9.8 × 10^8^	7.6 × 10^8^	17A	0	0
01G	2.68 × 10^6^	2.51 × 10^6^	10B	2.84 × 10^8^	4.2 × 10^7^	17B	3.3 × 10^7^	1.8 × 10^7^
01H	4.7 × 10^7^	0	11A	2.12 × 10^6^	9.7 × 10^5^	18A	5 × 10^7^	1.2 × 10^7^
01I	9.5 × 10^8^	8.9 × 10^8^	11B	0	0	18B	1 × 10^6^	1.9 × 10^4^
02A	5.7 × 10^7^	4.67 × 10^6^	12A	2.02 × 10^6^	1.15 × 10^5^	19A	1.05 × 10^5^	0
02B	1.82 × 10^8^	1.41 × 10^8^	13A	0	0	19B	9.3 × 10^8^	0
03A	9.7 × 10^7^	7.5 × 10^7^	13B	1 × 10^7^	0	19C	1.2 × 10^7^	5.9 × 10^6^
03B	2.41 × 10^9^	2.1 × 10^9^	13C	2 × 10^6^	0	19D	1.02 × 10^7^	1 × 10^5^
03C	5 × 10^6^	0	13D	3 × 10^7^	0	19E	9.4 × 10^8^	8.9 × 10^8^
03D	6 × 10^4^	5.7 × 10^4^	14A	1.9 × 10^7^	4.1 × 10^6^	19F	0	0
03E	1.82 × 10^7^	8.4 × 10^6^	14B	7 × 10^7^	2.9 × 10^6^	20A	2.89 × 10^8^	3.3 × 10^6^
04A	8.7 × 10^5^	0	15A	2.6 × 10^8^	8.2 × 10^7^	20B	8.2 × 10^7^	1 × 10^6^
05A	6.1 × 10^6^	5.5 × 10^7^	16A	9.2 × 10^8^	0	—	—	—
06A	9.3 × 10^7^	4.3 × 10^5^	16B	9.41 × 10^7^	0	—	—	—

**Table 2:** Correlation microbial contaminants with different herbal preparations in Gondar Town, Northwest Ethiopia, from April 1 to May 25, 2013.

Sample ID	Nature of sample/therapeutic claims	Scientific name	Isolated bacteria	Sample ID	Nature of sample/therapeutic claims	Scientific name	Isolated bacteria
01A	Liquid: antigastric & GI problem	ND	*Enterobacter cloacae*, *Citrobacter*	07A	Powder: antiabdominal problem	*Cucurbita maxima*	*Bacillus *spp. *S. epidermidis, Citrobacter diversus*

01B	Liquid: anticonstipation	ND	*No*	08A	Powder: antiliver infection	*Terminalia schimperiana*	*Providencia stuartii*

01C	Powder: antihypertension & fungal infection	*Dodonaea angustifolia*	*Enterobacter aerogenes*, *Bacillus *spp., *P. aeuroginosa*	08B	Powder: evil eye	*Myrica salicifolia*	*E. coli*, *Bacillus *spp. *Providencia*

01D	Powder: anti-heliments & sinus	*Cucurbita maxima*	*S. aureus*, *S. dysenteriae*, *Providencia stwarti*	09A	Liquid: antiwound & fungal infection	*Plumbago zeylanicum*	*Bacillus *spp. &* Enterobacter cloacae*

01E	Powder: antiwound infection	*Plumbago zeylanicum*	*Bacillus *spp.	09B	Powder: antiabdominal problem	ND	*Citrobacter diversus*, *E. coli*, *S. dysenteriae, Bacillus *spp.

01F	Powder: anti-HIV	*Foeniculum vulgare*	*S. pyogen*	10A	Powder: anti-UTI infection	*Clerodendrum myricoides*	*Enterobacter cloacae*, *K. pneumonia*

01G	Powder: effective for menstrual abnormality	ND	*Enterobacter cloacae*, *Citrobacter*, *Acinetobacter* spp., *S. dysenteriae*	10B	Powder: acts as pellagra	ND	*Providencia,Citrobacter*, *K. ozaenae, Bacillus *spp.

01H	Powder: effective against warts & bleeding	*Calotropis procera*	*Bacillus *spp.	11A	Powder: antigastric	ND	*Bacillus *spp., *S. aureus, Pseudomonas aeruginosa*

01I	Liquid: antiotitis media	*Withania somnifera*	*Salmonella *spp., *S. dysenteriae*, *E. coli,* *K. pneumoniae*	11B	Powder: antiliver disease	ND	*No growth *

02A	Powder: effective for kidney and liver infections	Jestica schimperiana	*Enterobacter cloacae*, *Serratia, Bacillus *spp.	12A	Liquid: effective for otitis media	*Withania somnifera*	*S. aureus*, *S. pyogen*, *K. pneumonia*, *enterococci*, *K. ozaenae *

02B	Powder: effective for kidney infections	*Plumbago zelylanicum*	*Enterobacter cloacae*, *Acinetobacter*, *Bacillus *spp., *E. coli*	13A	Liquid: swollen wound	*Brucea antidysenterica*	*No growth *

03A	Powder: antiwart & wound	ND	*Enterobacter cloacae & K. ozaenzae*	13B	Powder: wound infection	*Clerodendrum myricoides*	*S. aureus, Bacillus *spp.

03B	Powder: antiasthma	*Rumex abyssinicus*	*Bacillus *spp., *S. aureus*, * P. aurognosa* *Acinetobacter *spp.	13C	Powder: effective against liver disease	*Thymus schimperi*	*Bacillus *spp*., S. epidermidis*

03C	Powder: antiscar	ND	*Bacillus *spp.	13D	Powder: rabbis virus	ND	*S. saprophyticus *&* Bacillus *spp.

03D	Liquid: antifungal	Dodonaea amgustifolica	*Serratia*	14A	Powder: antiepilepsy	ND	*S. aureus, Bacillus *spp., *K. pneumoniae*, *Citrobacter diversus *

03E	Powder: antifungal & skin disease	*Brucea antidysenterica*	*Providencia stuartii*, *E. coli*, *Serratia, Salmonella *spp., *S. dysenteriae*	14B	Powder: anticancer	*Plumbago zeylanicum* & ND	*Serratia*, *E. coli*, *S. dysenteriae, Bacillus *spp.

04A	Powder: antiwart	*Cucumis prophetarum*	*Bacillus *spp.	15A	Powder: antiparalysis	ND	*E. coli*, *K. pneumonia*, *Enterobacter aerogenes*, *Salmonella *spp*., S. epidermidis*

05A	Powder: antiwart	*Plumbago zeylanicum*	*E. coli*, *S. dysenteriae, Enterobacter *	16A	Liquid: antidiarrheal	ND	*S. pyogenes* &* Bacillus *spp.

06A	Powder: acts as pellagra	*Aloe vera*	*Enterobacter cloacae*, *S. aureus*, *S. epidermidis*, *K. pneumonia*	16B	Liquid: anti-intestinal parasites	*Oxalis semiloba*	*S. pyogen*

16C	Powder: anti-intestinal parasites	ND	*Enterobacter cloacae*	18B	Powder: antiherpes zoster virus	ND	*S. saprophyticus*, *K. pneumonia*

16D	Powder: anti-intestinal parasites & food poisoning	ND	*K. pneumonia*, *S. dysenteriae*	19A	Liquid: antirash	ND	*Bacillus *spp.

16E	Powder: antisinus & nose asthma	*Myrica salicifolia*	*S. epidermidis*, *Bacillus *spp.	19B	Liquid: antiepilepsy	ND	*Bacillus *spp.

16F	Liquid: antiwound infection	*Clerodendrum myricoides*	*Bacillus *spp. *Enterobacter cloacae*	19C	Powder: antimalaria intestinal parasites & cancer	*Croton macrostachyus*	*S. dysenteriae*, *K. pneumonia *

16G	Powder: antiwound infection	*Clerodendrum myricoides*	*S. dysenteriae*, *Bacillus *spp.	19D	Powder: antiwound	*Clerodendrum myricoides*	*S. aureus*, *S. dysenteriae*

17A	Powder: antiwound infection & diarrhea	ND	No growth	19E	Powder: antifungal	Aloe vera	*K. pneumonia*, *Providencia stuarti*, *Serratia*

17B	Powder: antigastric	ND	*S. aureus*, *K. pneumonia,* *S. dysenteriae*	19F	Liquid: antiotitis media	*Withania somnifera*	No growth

18A	Powder: anti-intestinal parasites & burns	ND	*Shigella dysenteriae*, *E. coli *	20A	Powder: antiepilepsy	ND	*Citrobacter, Bacillus *spp.

				20B	Powder: antidiabetes & MTB	ND	*E. coli*, *Bacillus *spp.

ND: not yet determined, ID: identification number, the same number or ID indicates a single herbalist and spellings in front of numbers indicate different herbal medicines of the same herbalist.

**Table 3:** Bacterial profiles isolated from herbal medicinal products sold in different markets in Gondar Town, Northwest Ethiopia, from April 1 to May 25, 2013.

Bacterial isolates	Frequency *N* (%)
*Gram negatives*	
*Enterobacter cloacae*	18 (12)
*Enterobacter aerogenes *	4 (2.7)
*Shigella dysenteriae*	13 (8.7)
*Klebsiella pneumoniae *	11 (7.3)
*Klebsiella ozaenae *	4 (1.7)
*Escherichia coli *	10 (6.7)
*Providencia *spp.	12 (8.0)
*Citrobacter *spp.	12 (8.0)
*Serratia *spp.	5 (3.3)
*Acinetobacter* spp.	4 (2.7)
*Salmonella *spp.	3 (2.0)
*Pseudomonas aeruginosa*	2 (1.3)
*Gram positives*	
*Bacillus* spp.	31 (20.7)
*Staphylococcus aureus *	10 (6.7)
*Staphylococcus epidermidis *	5 (3.3)
*Staphylococcus saprophyticus*	2 (1.3)
*Streptococcus pyogenes *	4 (2.7)

*Total*	*150 (100%)*

**(a):** 

Bacterial isolate	Total number	Pattern	AMP number (%)	AML number (%)	AMC number (%)	SXT number (%)	CN number (%)	F number (%)	TE number (%)	C number (%)	CRO number (%)	N number (%)	CIP number (%)
*Enterobacter cloacae *	18	S	0 (0)	7 (38.8)	5 (27.7)	15 (83.3)	17 (94.4)	8 (44.4)	15 (83.3)	15 (83.3)	12 (66.6)	16 (88.8)	15 (83.3)
		R	18 (100)	11 (61.1)	13 (72.2)	3 (16.6)	1 (5.4)	10 (55.5)	3 (16.6)	3 (16.6)	6 (33.3)	2 (11.1)	3 (16.6)
*Shigella dysenteriae*	13	S	2 (15.4)	3 (23)	7 (53.8)	12 (92.3)	12 (92.3)	7 (53.8)	13 (100)	13 (100)	12 (92.3)	12 (92.3)	12 (92.3)
		R	11 (84.6)	10 (76.9)	6 (46.1)	1 (7.7)	1 (7.7)	6 (46.1)	0 (0)	0 (0)	1 (7.7)	1 (7.7)	1 (7.7)
*Klebsiella pneumonia*	11	S	0 (0)	2 (18.2)	5 (45.5)	8 (72.7)	10 (90.9)	6 (54.5)	9 (81.8)	10 (90.9)	7 (63.6)	11 (100)	10 (90.9)
		R	11 (100)	9 (81.8)	6 (54.5)	3 (27.3)	1 (9.1)	5 (45.5)	2 (18.2)	1 (9.1)	4 (36.4)	0 (0)	1 (9.1)
*Escherichia coli*	10	S	2 (20)	4 (40)	7 (70)	9 (90)	10 (100)	5 (50)	10 (100)	8 (80)	10 (100)	9 (90)	10 (100)
		R	8 (80)	6 (60)	3 (30)	1 (10)	0 (0)	5 (50)	0 (0)	2 (20)	0 (0)	1 (10)	0 (0)
*Citrobacter diversus*	9	S	1 (11.1)	7 (77.8)	6 (66.7)	9 (100)	9 (100)	5 (55.6)	9 (100)	9 (100)	8 (88.9)	9 (100)	8 (88.9)
		R	8 (88.9)	2 (22.2)	3 (33.3)	0 (0)	0 (0)	4 (44.4)	0 (0)	0 (0)	1 (11.1)	0 (0)	1 (11.1)
*Providencia stuartii*	8	S	0 (0)	6 (75)	1 (12.5)	8 (100)	8 (100)	1 (12.5)	7 (87.5)	8 (100)	6 (75)	7 (87.5)	7 (87.5)
		R	8 (100)	2 (25)	7 (87.5)	0 (0)	0 (0)	7 (87.5)	1 (12.5)	0 (0)	2 (75)	1 (12.5)	1 (12.5)
*Serratia *	5	S	3 (60)	4 (80)	4 (80)	5 (100)	5 (100)	3 (60)	4 (80)	5 (100)	5 (100)	5 (100)	5 (100)
		R	2 (40)	1 (20)	1 (20)	0 (0)	0 (0)	2 (40)	1 (20)	0 (0)	0 (0)	0 (0)	0 (0)
*Klebsiella ozaenae*	4	S	1 (25)	2 (50)	2 (50)	3 (75)	3 (75)	0 (0)	2 (50)	2 (50)	3 (75)	4 (100)	4 (100)
		R	3 (75)	2 (50)	2 (50)	1 (25)	1 (25)	4 (100)	2 (50)	2 (50)	1 (75)	0 (0)	0 (0)
*Enterobacter aerogenes *	4	S	0 (0)	2 (50)	1 (25)	3 (75)	4 (100)	1 (25)	3 (75)	4 (100)	2 (50)	3 (75)	3 (75)
		R	4 (100)	2 (50)	3 (75)	1 (25)	0 (0)	3 (75)	1 (25)	0 (0)	2 (50)	1 (25)	1 (25)
*Providencia*	4	S	1 (25)	1 (25)	1 (25)	4 (100)	4 (100)	1 (25)	4 (100)	3 (75)	3 (75)	3 (75)	3 (75)
		R	3 (75)	3 (75)	3 (75)	0 (0)	0 (0)	3 (75)	0 (0)	1 (25)	1 (25)	1 (25)	1 (25)
*Acinetobacter *	4	S	0 (0)	1 (25)	1 (25)	4 (100)	3 (75)	0 (0)	4 (100)	2 (50)	2 (50)	4 (100)	4 (100)
		R	4 (100)	3 (75)	3 (75)	0 (0)	1 (25)	4 (100)	0 (0)	2 (50)	2 (50)	0 (0)	0 (0)
*Salmonella* spp.	3	S	0 (0)	0 (0)	2 (66.7)	3 (100)	3 (100)	2 (66.7)	3 (100)	3 (100)	3 (100)	3 (100)	3 (100)
		R	3 (100)	3 (100)	1 (33.3)	0 (0)	0 (0)	1 (33.3)	0 (0)	0 (0)	0 (0)	0 (0)	0 (0)
Citrobacter	3	S	0 (0)	2 (66.7)	0 (0)	3 (100)	3 (100)	2 (66.7)	3 (100)	3 (100)	2 (66.7)	3 (100)	3 (100)
		R	3 (100)	1 (33.3)	3 (100)	0 (0)	0 (0)	1 (33.3)	0 (0)	0 (0)	1 (33.3)	0 (0)	0 (0)
*Pseudomonas aeruginosa *	2	S	0 (0)	0 (0)	0 (0)	2 (100)	1 (50)	0 (0)	1 (50)	2 (100)	1 (50)	2 (100)	2 (100)
		R	2 (100)	2 (100)	2 (100)	0 (0)	1 (50)	2 (100)	1 (50)	0 (0)	1 (50)	0 (0)	0 (0)

AMP: ampicillin, AML: amoxacillin, AMC: amoxicilin + clavulanic acid, SXT: cotrimoxazole, CN: gentamicin, F: nitrofurantoin, TE: tetracycline, C: chloramphenicol, CRO: ceftriaxone, N: norfloxacin, and CIP = ciprofloxacin.

**(b):** 

Bacterial isolate	Total number	Pattern	AMP *N* (%)	AML *N* (%)	AMC *N* (%)	SXT *N* (%)	CN *N* (%)	F *N* (%)	TE *N* (%)	C *N* (%)	CRO *N* (%)	N *N* (%)	CIP *N* (%)	CXC *N* (%)	PEN *N* (%)	ERY *N* (%)	V *N* (%)
*Bacillus *spp.	31	S	3 (10)	16 (52)	5 (26)	29 (93.5)	31 (100)	21 (68)	30 (97)	30 (97)	22 (71)	16 (94)	31 (100)	14 (45.2)	12 (38.7)	24 (77.4)	23 (74.2)
		R	28 (90)	15 (48)	23 (74)	2 (6.5)	0 (0)	10 (32)	1 (3.2)	1 (3.2)	9 (29)	2 (6.5)	0 (0)	17 (54.8)	19 (61.3)	7 (22.6)	8 (25.8)
*Staphylococcus aureus*	10	S	2 (20)	6 (60)	6 (60)	10 (100)	10 (100)	6 (60)	9 (90)	10 (100)	9 (90)	10 (100)	10 (100)	7 (70)	4 (40)	10 (100)	10 (100)
		R	8 (80)	4 (40)	4 (40)	0 (0)	0 (0)	4 (40)	1 (10)	0 (0)	1 (10)	0 (0)	0 (0)	3 (30)	6 (60)	0 (0)	0 (0)
*Staphylococcus epidermidis*	5	S	1 (20)	3 (60)	2 (40)	3 (60)	5 (100)	5 (100)	5 (100)	5 (100)	3 (60)	4 (80)	4 (80)	5 (100)	2 (40)	3 (60)	5 (100)
		R	4 (80)	2 (40)	3 (60)	2 (40)	0 (0)	0 (0)	0 (0)	0 (0)	2 (40)	1 (20)	1 (20)	0 (0)	3 (60)	2 (40)	0 (0)
*Staphylococcus saprophyticus*	2	S	0 (0)	2 (100)	2 (100)	2 (100)	2 (100)	1 (50)	2 (100)	2 (100)	1 (50)	2 (100)	2 (100)	0 (0)	2 (100)	2 (100)	2 (100)
		R	2 (100)	0 (0)	0 (0)	0 (0)	0 (0)	1 (50)	0 (0)	0 (0)	1 (50)	0 (0)	0 (0)	2 (100)	1 (50)	0 (0)	0 (0)
*Streptococcus pyogenes *	4	S	3 (75)	3 (75)	4 (100)	3 (75)	3 (75)	3 (75)	4 (100)	4 (100)	2 (50)	3 (75)	3 (75)	3 (75)	1 (50)	0 (0)	4 (100)
		R	1 (25)	1 (25)	0 (0)	1 (25)	1 (25)	1 (25)	0 (0)	0 (0)	2 (50)	1 (25)	1 (25)	1 (25)	1 (25)	0 (0)	0 (0)

AMP: ampicillin, CAF: chloramphenicol, N: norfloxacin, AML: amoxacillin, CIP: ciprofloxacin, SXT: cotrimoxazole, CRO: ceftriaxone, CN: gentamicin, TTC: tetracycline, AMC: amoxicilin + clavulanic acid, CXC: cloxacillin, PEN: penicillin, ERY: erythromycin, and V: vancomycin.

**Table 5:** Drug resistant patterns of bacterial isolates identified from herbal medicinal products in Gondar, Northwest Ethiopia, from April 1 to May 25, 2013.

List of antibiotics	Rate of resistance among gram negatives *N* = 98 (%)	Rate of resistance among gram positives *N* = 52 (%)	Total *N* = 150 (%)
Cotrimoxazole	10 (10.2)	5 (9.6)	15 (10%)
Gentamycin	6 (6.1)	1 (1.9)	7 (4.7%)
Tetracycline	11 (11.2)	2 (3.8)	13 (8.7%)
Nitrofurantoin	57 (58.2)	16 (30.8)	73 (48.7%)
Amoxicillin clavulanic acid	65 (63.3)	30 (42.3)	87 (63.3%)
Ceftriaxone	22 (22.4)	16 (30.8)	38 (25.4%)
Ampicillin	88 (89.8)	43 (82.7)	131 (87.3%)
Amoxicillin	58 (59.8)	34 (65.4)	92 (61.3%)
Chloramphenicol	11 (11.2)	1 (1.9)	12 (8%)
Norfloxacin	7 (7.1)	4 (7.6)	11 (7.3%)
Ciprofloxacin	9 (9.2)	2 (3.8)	11 (7.3%)
Cloxacillin	—	23 (44.2)	23 (15.3%)
Penicillin	—	30 (57.7)	30 (20.0%)
Erythromycin	—	9 (17.3)	9 (6.0%)
Vancomycin	—	8 (15.4)	8 (5.3%)

**Table 6:** Multidrug resistance patterns of bacterial isolates from herbal medicinal products sold in Gondar Town, Northwest Ethiopia, 2013.

Bacterial isolates	Total (%)	Number of antibiotics resistance patterns *N* (%)					
		*R*0	*R*1	*R*2	*R*3	*R*4	≥*R*5
*Gram negatives*	*98 (65.3)*	*7 (7.1)*	*9 (9.2)*	*19 (19.4)*	*22 (22.5)*	*19 (19.4)*	*22 (22.4)*
* Enterobacter cloacae*	18 (18.4)	0 (0)	0 (0)	4 (22.2)	5 (27.7)	5 (27.7)	4 (22.2)
* Enterobacter aerogenes *	4 (4)	0 (0)	0 (0)	0 (0)	2 (50)	1 (25)	1 (25)
* Shigella dysenteriae *	13 (13.3)	2 (15.4)	1 (7.7)	2 (15.4)	2 (15.4)	5 (38.5)	1 (7.7)
* Klebsiella pneumonia *	11 (11.2)	0 (0)	1 (9.1)	3 (27.3)	3 (27.3)	0 (0)	4 (36.4)
* Klebsiella ozaenae *	4 (4)	0 (0)	1 (25)	1 (25)	0 (0)	0 (0)	2 (50)
* Escherichia coli*	10 (10.2)	2 (20)	1 (10)	3 (30)	1 (10)	1 (10)	2 (20)
* Providencia stuartii*	12 (12.2)	1 (8.3)	0 (0)	1 (8.3)	3 (25)	4 (33.3)	3 (25)
* Citrobacter *spp.	12 (12.2)	1 (8.3)	3 (25)	2 (16.7)	2 (16.7)	3 (25)	0 (0)
* Serratia *	5 (5.1)	1 (20)	2 (40)	1 (20)	1 (20)	0 (0)	0 (0)
* Salmonella *spp.	3 (3)	0 (0)	0 (0)	1 (33.3)	2 (66.7)	0 (0)	0 (0)
* Pseudomonas aeruginosa *	2 (2)	0 (0)	0 (0)	0 (0)	0 (0)	0 (0)	2 (100)
* Acinetobacter*	4 (4)	0 (0)	0 (0)	0 (0)	1 (25)	0 (0)	3 (75)
*Gram positives*	*52 (34.7)*	*7 (13.5)*	*2 (3.8)*	*4 (7.7)*	*12 (23.1)*	*9 (17.3)*	*18 (34.6)*
* Bacillus *spp.	31 (59.6)	4 (12.9)	1 (3.2)	4 (12.9)	4 (12.9)	7 (22.6)	11 (35.5)
* Staphylococcus aureus *	10 (19.2)	1 (10)	0 (0)	0 (0)	5 (50)	1 (10)	3 (30)
* Staphylococcus epidermidis*	5 (9.6)	1 (20)	0 (0)	0 (0)	2 (40)	0 (0)	2 (40)
* Staphylococcus saprophyticus*	2 (3.8)	0 (0)	1 (50)	0 (0)	0 (0)	1 (50)	0 (0)
* Streptococcus pyogenes*	4 (7.7)	1 (25)	0 (0)	0 (0)	1 (25)	0 (0)	2 (50)

*Total MDR (G− and G+)*	*150 (100)*	*14 (9.3)*	*11 (7.3)*	*23 (15.3)*	*34 (22.7)*	*28 (18.7)*	*40 (26.7)*

MDR: multiple drug resistance (resistant to 2 or more antibiotics), *R*0: no antibiotic resistance, *R*1: resistance to one, *R*2: resistance to two, *R*3: resistance to three, *R*4: resistance to four, and ≥*R*5: resistance to five and more drugs.
